# Supplementary figures and images for: Ultra-performance liquid chromatography-mass spectrometry for precise fatty acid profiling of oilseed crops
Source: PeerJ. 2019 Mar 6;7:e6547. doi: 10.7717/peerj.6547 (PMC6408914; doi:10.7717/peerj.6547)

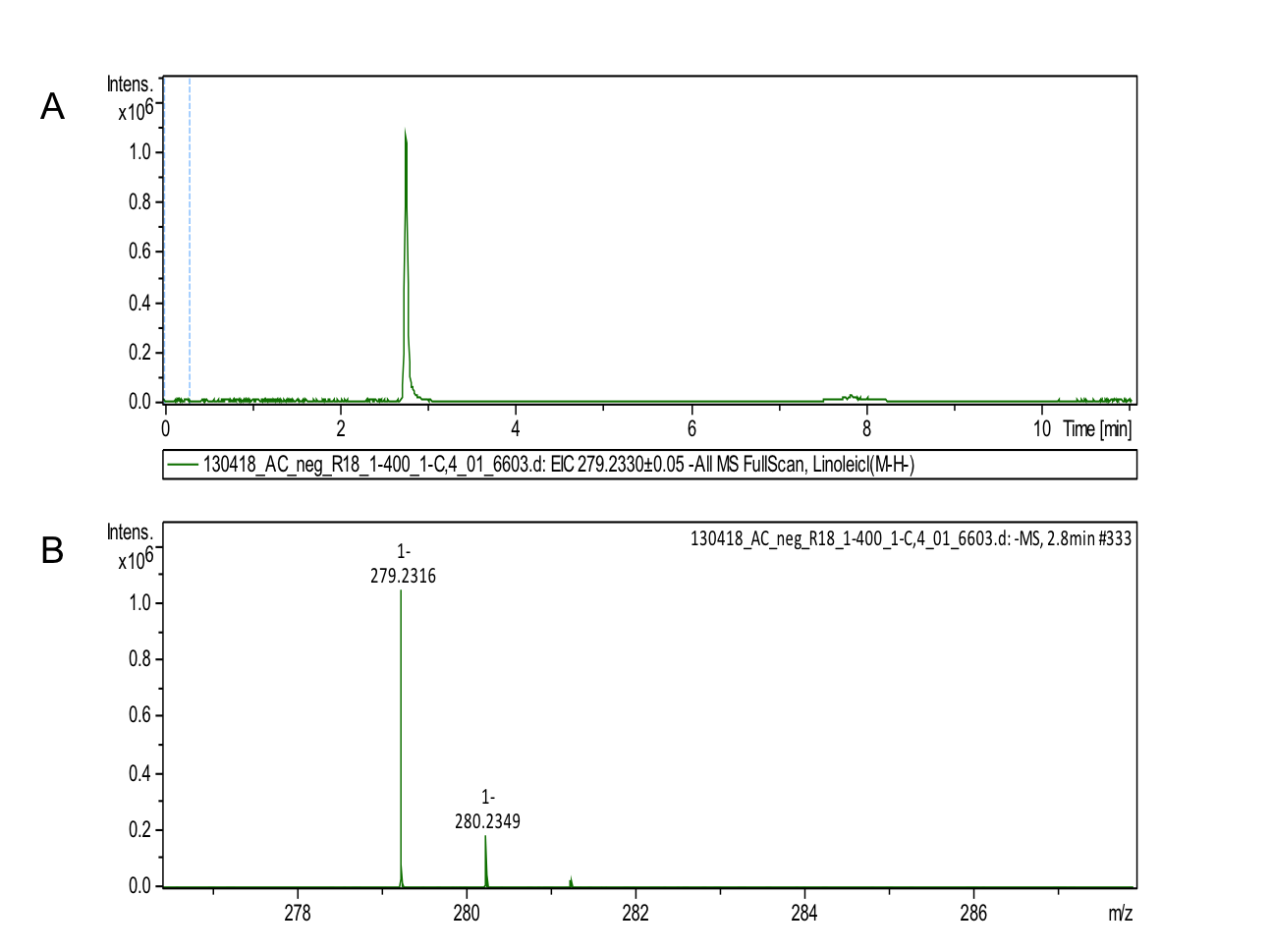

Supplement: Figure S1 — A. Extracted ion chromatogram for 18:1 FA B. MS spectrum for 18:1 FA with 3 isotope peaks. X-axis -retention time in min, Y-axis -intensity in counts. [file peerj-07-6547-s004.png]

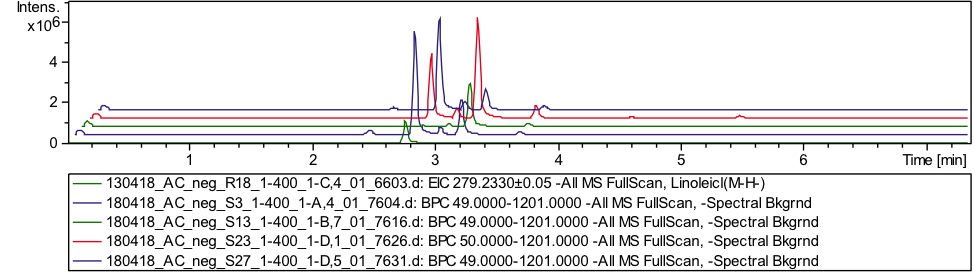

Supplement: Figure S2 — The abundance of major FAs vary between sunflower samples measured by UPLC-5. Each colored line -chromatogram of 1 sample. X-axis -retention time in min, Y-axis -intensity in counts. [file peerj-07-6547-s005.png]
